# Influence of scaler tip design on root surface roughness, tooth substance loss and patients’ pain perception: an in vitro and a randomised clinical trial

**DOI:** 10.1186/s12903-021-01540-0

**Published:** 2021-03-31

**Authors:** Nur Ayman Abdul Hayei, Noor Azlin Yahya, Syarida Hasnur Safii, Roslan Saub, Rathna Devi Vaithilingam, Nor Adinar Baharuddin

**Affiliations:** 1 Department of Restorative Dentistry, Faculty of Dentistry, University of Malaya, Lembah Pantai, 50603 Kuala Lumpur, Malaysia; 2 Department of Community Oral Health and Clinical Prevention, Faculty of Dentistry, University of Malaya, Lembah Pantai, 50603 Kuala Lumpur, Malaysia; 3 Faculty of Dentistry, Universiti Sains Islam Malaysia, Pandan Indah, 56100 Kuala Lumpur, Malaysia

**Keywords:** Pain perception, Scaler tip design, Tooth surface roughness, Tooth substance loss, Ultrasonic scaler

## Abstract

**Background:**

The influence of scaler tip design on root surface roughness, tooth substance loss and patients’ pain perception is investigated.

**Methods:**

This article was divided into the following parts: Part 1 Surface roughness and substance loss: an in vitro study, which involves intact extracted teeth sectioned and treated using a piezoelectric ultrasonic device (PM200 EMS Piezon, Switzerland) with a conventional scaler tip (FS-407) and a Perio Slim (PS) scaler tip (Perio Slim DS-016A). All sectioned samples for tooth surface roughness (n = 20) and tooth substance loss (n = 46) analyses were measured and compared using a 3D surface texture analyser and scanning electron microscope (SEM) respectively, at baseline and following scaling. Part 2 Pain Perception: a clinical study, which was a split mouth study design including 30 participants with gingivitis and/or mild chronic periodontitis; treated with supra-gingival scaling from teeth #13 to #23. Subjects were randomised to group A or group B. Group A was treated first with PS scaler tips, whereas group B was treated first with conventional scaler tips. Pain perception was recorded using the visual analogue scale (VAS).

**Results:**

In vitro study: both scaler tips caused significant reduction in root substance roughness after scaling (*p* < 0.05), but no significant difference between the two scaler tips (*p* > 0.05) was observed. The PS scaler tip caused statistically significantly less root substance loss (*p* < 0.05) when the initial thickness of the tooth was < 1000 µm. Clinical study: the participants reported significantly lesser pain score during scaling using the PS scaler tip (median: 3) than when using the conventional scaler tip (median: 5) (*p* < 0.05).

**Conclusions:**

In the in vitro study, using a slim scaler tip design causes less tooth substance loss compared to a wider scaler tip design. In the clinical study, less pain was observed compared than a wide (conventional) scaler tip design.

## Background

The effective removal of calculus and prevention of the recolonization of periodontal bacteria to periodontal pockets are common strategies used in periodontal therapy [1]. Hand instruments are used first in removing calculus, but using them is time-consuming [2, 3]. To remove calculus effectively, powered devices, such as ultrasonic scalers, have been introduced as alternatives to hand instruments. For decades, ultrasonic scalers have been widely used, but the possibility that their use causes damage to the root surface and discomfort to patients has caused concern.

The performance of an ultrasonic scaler increases with displacement amplitude, and the magnitude of displacement amplitude increases when the power setting is increased, working load is reduced, and a wide scaler tip is used [4, 5]. Displacement amplitude is highly sensitive and varies among different generators [4].

Tooth substance loss as a result of a scaling procedure with an ultrasonic scaler produces a rough surface that facilitates the recolonization of bacteria on the root surface [6]. Excessive cementum substance removal may lead to exposed dentinal tubules and root sensitivity [7]. The assessment of root surface roughness on the basis of the comparison among piezoelectric ultrasonic and magnetostrictive scalers demonstrated that piezoelectric ultrasonic scaler tip produces rougher surfaces than magnetostrictive scaler tip [3, 8].

A new-generation piezo ultrasonic scaler designed for gentle biofilm removal, Vector ™ (Durr Dental), was introduced in year 2000. Vector produces smoother root surfaces than conventional hand scaler [9]. This scaler tip produces linear oscillation with low-displacement amplitude for reducing impact on tooth surface. Thus, this highlighted the importance of linear oscillation with low-displacement amplitude on the reduction of root surface roughness. Ultrasonic scaler with slim scaler tip design produced elliptical oscillation pattern, but during scaling, the loading will dampen the displacement amplitude and produced linear oscillation pattern [10].

The analysis of tooth substance loss after scaling suggests that ultrasonic scalers remove lesser amount of tooth substance than hand instruments [9, 11, 12]. Furthermore, Jepsen et al. [13] reported that the amount of tooth substance loss was significantly lower when a narrow type scaler tip design than when a wider tip was used.

Patient’s compliance to dental treatment is affected by several factors, including socioeconomic status, dental fear and dentist’s behaviour [14]. According to a survey by Berggren and Meynert, painful dental work is the most commonly mentioned reason for fear in dentistry among adults [15, 16]. Therefore, delivering dental treatment with minimal pain can positively affect patients’ compliance and improve treatment success. New scaler tip designs have been developed to reduce patients’ discomfort during scaling. Braun et al. [17] reported that a slim-type scaler tip can reduce pain sensation during scaling compared with a conventional tip. However, the influence of scaler tip design on patient’s pain perception has not been thoroughly investigated. Therefore, the aim of this study was to compare the effect of Perio Slim and conventional scaler tips during ultrasonic scaling related to three aspects: tooth surface roughness, tooth substance loss and patient’s pain perception.

## Methods

This section describes the methodology in two parts: Part 1 Surface roughness and substance loss—an in vitro study that investigated the influence of scaler tip design on tooth surface roughness and tooth substance loss, and Part 2 Pain perception—a clinical study that investigated patients’ pain perception.

### Part 1: surface roughness and substance loss: an in vitro study

Single-rooted human permanent teeth extracted within the last 6 months were obtained from a government dental clinic in Bangsar, Kuala Lumpur. The teeth were disinfected using 0.5% chloramine-T trihydrate (Across, Belgium) for a week. All teeth are then stored in distilled water, and placed in a fridge at 4 °C before analysis. The inclusion criteria for tooth selection were as follows: (1) sound teeth and (2) minimal calculus. The exclusion criteria were (1) teeth with crack lines; (2) teeth with signs of abrasion and/or erosion; and (3) hypomineralised teeth, or teeth with signs of amelogenesis imperfecta and dentinogenesis imperfecta.

**Fig. 1:**
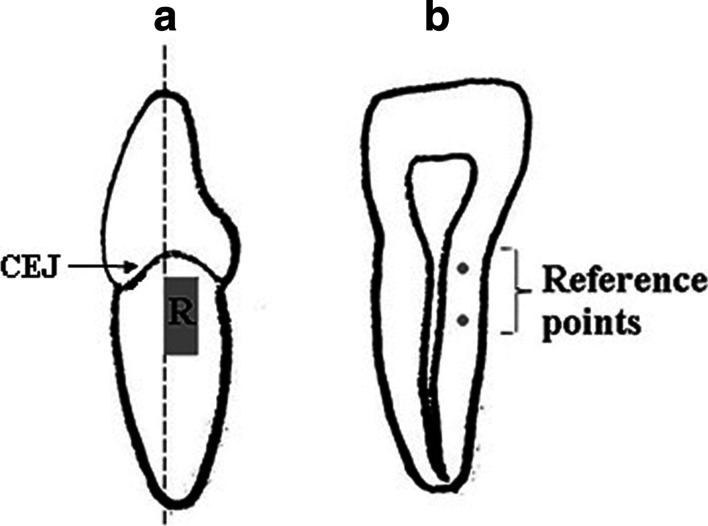
Diagram showing tooth preparation. **a** The mesial view of an incisor sectioned indicated by dotted line. An area of 3 mm × 5 mm area was marked apical to CEJ for roughness assessment, as indicated by R. Scaling was performed at R. **b** The cross-sectional view of the incisor tooth showing two reference points marked using a scaler tip

Before the experiment, the teeth were prepared as follows: Approximately 3 mm of the apical portion of each tooth was embedded in a clear cold-curing epoxy resin (Mirapox®, Balakong, Malaysia) for subsequent cutting. The tooth was sectioned in the apico-coronal direction with a slow-speed precision cutter (Metkon, Bursa, Turkey). Apical to the CEJ by 1 mm, an area of 3 mm (width) × 5 mm (height) was marked using a permanent marker (Fig. 1a).

Two indentations, both 1 mm in depth, were made approximately 1 to 2 mm from the outer tooth surface with a scaler tip, which correlates to the area for surface roughness assessment. The indentations represented upper and lower reference points, as shown in Fig. 1b. These reference points were used in measuring the amount of tooth substance loss.

### Scaling procedure

**Fig. 2:**
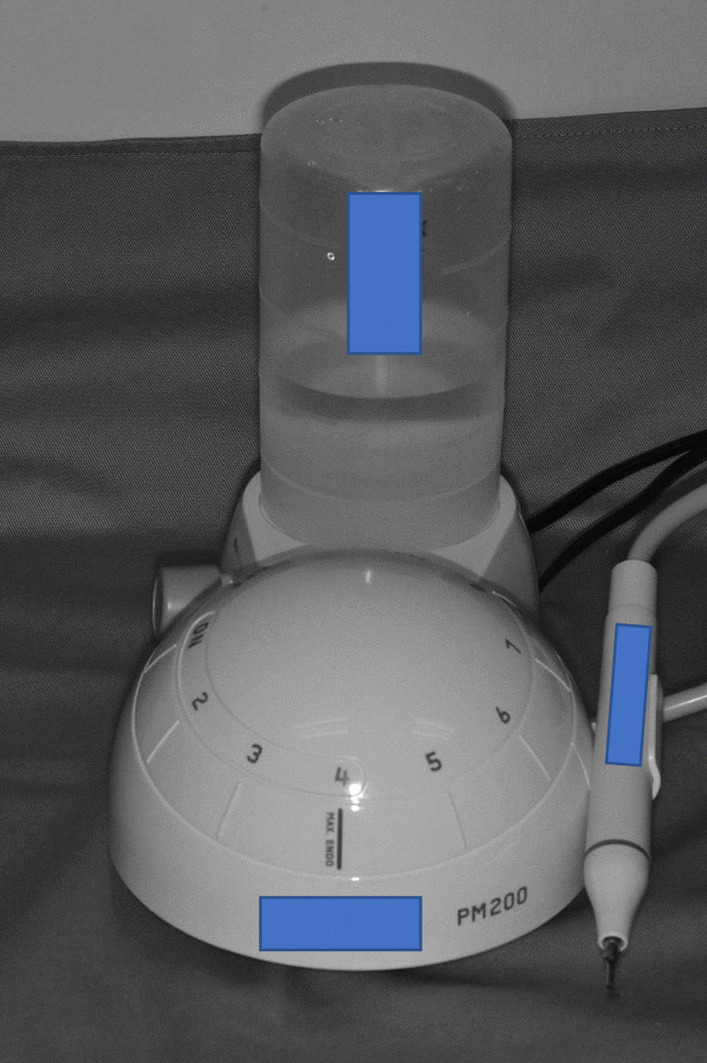
Portable ultrasonic scaler unit (PM200, EMS Switzerland)

**Fig. 3:**
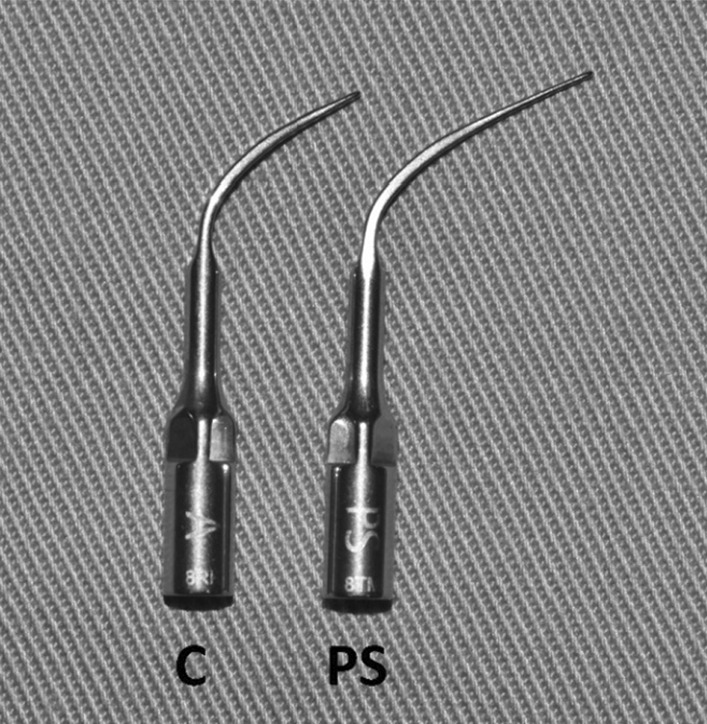
Lateral view of conventional (C) and Perio Slim (PS) scaler tips (EMS Piezon, Switzerland)

**Fig. 4:**
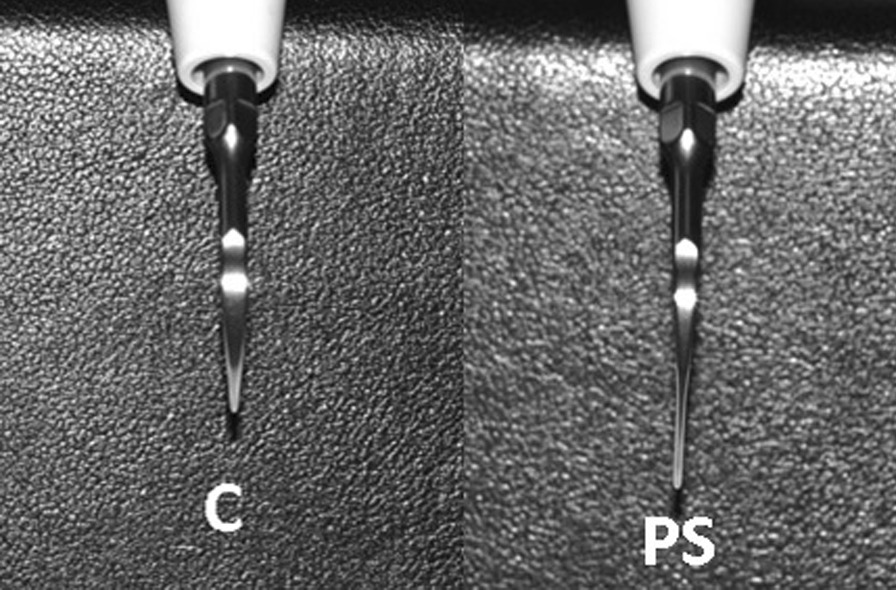
Frontal view of conventional (C) and Perio Slim (PS) scaler tips (EMS Piezon, Switzerland)

A portable ultrasonic scaler device (PM200, EMS^®^, Switzerland) (Fig. 2) was used to scale the root surface, labelled as R with a scaler tip, either conventional (FS-407, EMS Piezon, Switzerland) or PS (DS-016A, EMS Piezon, Switzerland) (Figs. 3, 4). A medium power setting (power level: 4) and the maximum water coolant level were used as recommended by the manufacturer. Teeth scaled using the conventional scaler tip represent the control group, whereas teeth scaled using PS scaler tip represent the test group.

Scaling was performed according to a standard protocol as follows. The scaler tip was placed parallel to the tooth surface and at zero-degree angle during scaling. The ultrasonic machine was activated prior to scaling to avoid the heaviest vertical movements. Once activated, the scaling was carried out in three continuous strokes with light force. The light force used was practiced without activating the tool, by probing under a fingernail with the amount of force that causes no pain [18]. Before the actual study, the above mentioned scaling protocol was practised until the operator felt comfortable. The procedure was performed by a single operator (NAAH).

### Tooth surface roughness assessment

Scaled tooth specimens were dried and mounted on a glass slab by means of utility wax. The specimens were observed under a 3D optical surface texture analyser (Alicona, InfiniteFocus Real3D, Austria). Surface roughness was determined as mean roughness (S_a_), defined as the average peaks and valley distances from the centre line of an area. The magnification was set at × 200 and 80 µm working length. Measurements were done in triplicate for each sample before and after scaling. The mean S_a_ values were then calculated.

### Quantification of tooth substance loss

Each tooth specimen was mounted in a position whereby reference points faced outward. The specimen was scanned using a low-vacuum scanning electron microscope (Quanta- FEG 50, FEI, Germany) on the cross-section surface, which included upper and lower reference points. The shortest distance between reference points and outer tooth surface was measured in micrometres (µm) and referred to as ‘tooth thickness’. Tooth thickness was measured in triplicate, and the mean was obtained. Tooth substance loss was the difference in tooth thickness before and after scaling. Magnification was set at 50 × and working distance of 10.0 mm.

### Part 2: clinical study

#### Study design

This study is a single-centre, single-blinded, and randomised controlled clinical study conducted at a teaching institution. Ethical approval was obtained from the Medical Ethics Committee Faculty of Dentistry, University of Malaya DF RD1705/0022(U).

*Trial registration* This clinical study followed the Consolidation Standards of Reporting Trials Statement and was retrospectively registered on 10/11/2020 at ClinicalTrials.gov (No. NCT04623723).

#### Participants

The protocol of this study was previously described in detail elsewhere [19]. The participants were those who sought scaling treatment in the Primary Care Unit, Faculty of Dentistry, University of Malaya. Those who fulfilled the inclusion criteria were invited to participate in the study. The inclusion criteria were healthy patients aged 20–40 years who had anterior maxillary teeth from teeth #13 to #23. They were diagnosed with chronic gingivitis and/or mild chronic periodontitis and presented with bleeding on probing (BOP) and supragingival calculus from teeth #13 to #23. Exclusion criteria were smokers, patients who had dentinal hypersensitivity, crowns, large restorations, non-vital teeth, acute dental infections or cervical caries lesions involving teeth #13 to #23. Patients who were on long-term non-steroidal anti-inflammatory drug therapy, undergoing orthodontic treatment or using removable partial dentures involving teeth from teeth #13 to #23 were also excluded.

#### Intervention

Supragingival scaling was carried out from teeth #13 to #23 with a portable ultrasonic scaler device (EMS) with either a conventional (FS-407, EMS Piezon, Switzerland) or PS (DS-016A, EMS Piezon, Switzerland) scaler tips. A medium power setting (power: 4) and maximum water coolant level were used as recommended by the manufacturer. The scaler tips were held parallel to the long axis of the tooth during scaling. Light force was used for scaling as described in the in vitro part, for a-2 min duration.

#### Outcome measures

The primary outcome for this study was pain perception.

#### Patients’ pain perception

**Fig. 5:**
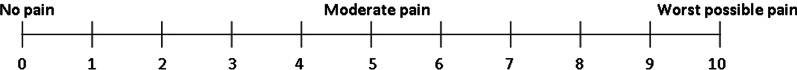
Visual Analogue Scale (VAS)

Pain perception was assessed using the Visual Analog Scale (VAS), which consists of a line numbered from 0 to 10. Scale ‘0’ indicates no pain, whereas scale ‘10’ indicates the worst possible pain. Detailed information about the procedure and how to score VAS were explained to all participants before the treatment. Participants were asked to complete the VAS score themselves by mark on the line the score that they feel represents their pain they experienced during treatment (Fig. 5). A new VAS registration paper was presented to each participant after each scaler tip change. This step prevented the participants from being influenced by their previous scores.

**Fig. 6:**
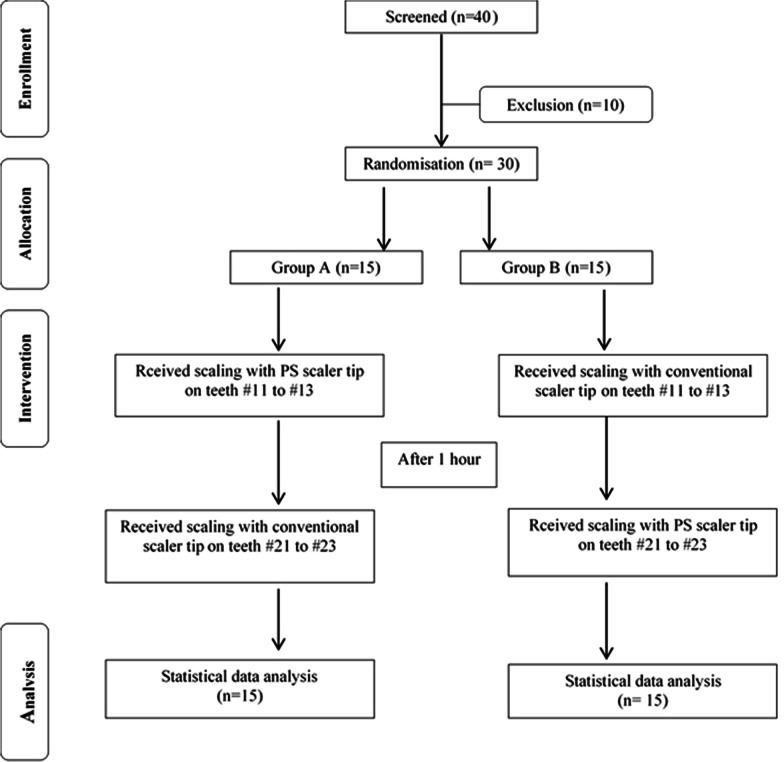
Consort 2010 flow chart of the clinical study

Data on socio-demography status, oral hygiene habits, education and lifestyle habits were collected using self-administered questionnaires. Periodontal parameters were recorded from teeth #13 to #23 by using a William’s probe (Hu-Friedy, Chicago, USA). The periodontal parameters measured were probing pocket depth (PPD), visible plaque index (VPI) (Ainamo & Bay 1975) and gingival bleeding index (GBI) (Ainamo & Bay 1975). The clinical examination was performed by a periodontics resident (NAAH) who was calibrated and trained by a periodontist. Figure 6 illustrates the CONSORT 2010 flow diagram of the study, graphically outlining the design and conduct of the clinical trial.

#### Sample size calculation

Data was analysed using G Power version 3 statistical software [20]. The sample size calculation was determined based on median difference of VAS score, 6.4 [U] in Braun et al. [17], the significance value was set at 0.05% and the power of the study was 80%. The estimated sample size was 25. Due to anticipated 20% dropout rate, thus a total 30 participants (15 participants per group) were recruited for this study.

#### Randomisation

Participants were recruited among individuals who were seeking periodontal treatment and matched the eligibility criteria. Randomisation was done with SPSS (Version 12.0.1, SPSS Inc., Chicago, USA) by a co-researcher (RS). For the removal of the ordering effect, predetermined recruitment numbers (1–30) were randomly allocated to groups A or B through generated randomisation list. The participants in group A received scaling with PS scaler tip first and then with a conventional scaler tip, whereas the participants in group B received scaling with a conventional scaler tip first and then with a PS scaler tip. The allocated groups were concealed in envelops.

#### Blinding

This study was a single-blinded study where each participant was blinded to the scaler tip design used. The double-blinded approach was not possible in this study because of some limitations: a single operator performed the intervention.

#### Data collection procedure

In the Primary Care Unit patients seeking scaling treatment were given appointments at the Periodontology Specialist Clinic, Faculty of Dentistry, University of Malaya. Appointment date was set, and screening for inclusion and exclusion criteria was performed. Clinical examination was performed. From 40 patients screened, 30 patients fulfilled the inclusion and exclusion criteria and were invited to participate in the study. Each participant was asked to fill out a questionnaire and was instructed to answer the VAS immediately after each scaling treatment procedure. The group allocation of the participants was only revealed to the operator after the clinical examination was done.

Each participant experienced scaling procedure using both scaler tips. The teeth were split into two sections: section 1 comprised teeth # 11 to # 13, and section 2 comprised teeth # 21 to #23. One of the sections was for the PS, and the other was for conventional scaler tips. The first round of scaling procedure for both groups started off with section 1 (teeth #13 to #11) using their respective scaler tips. The scaling procedure was performed for 2 min.

The second round of scaling was performed after an hour. Scaling was done on section 2 (teeth #21 to # 23) using the respective scaler tip. The same procedure was performed.

Immediately after each scaling procedure, the participants were asked to score their pain perception with the VAS. Full mouth scaling (if necessary) was given to subjects at the end of the study.

#### Data analyses

Data were analysed using SPSS statistical program (Version 16.0, SPSS Inc., Chicago, USA). Level of significance was set at *p* < 0.05. For the in vitro study, the normality of data was analysed using Shapiro–Wilk test. The intra-group comparison of tooth surface roughness (before and after) was analysed using Wilcoxon signed-rank test. Independent t test was used for inter-group comparison. For tooth substance loss, paired t test and independent t test were used in analysing data with normal distribution. For the clinical study, the sociodemographic comparison between groups was analysed using Mann–Whitney test. The baseline clinical parameters were analysed using paired sample t test. Based on Shapiro–Wilk test, VAS data distribution was not normal. Therefore, differences between the VAS scores after therapy of the two groups were compared using Wilcoxon signed-rank test.

## Results

### In vitro study

#### Tooth surface roughness

The mean surface roughness (Sa) value before scaling was 9.8 (± 4.7) µm and 10.0 (± 3.2) µm in PS and conventional groups, respectively (Table 1). After scaling, the Sa values were significantly reduced in both groups (*p* < 0.05). The reduction of surface roughness after scaling was 3.1 (± 2.5) µm and 4.7 (± 3.3) µm for PS and conventional group, respectively. However, the difference between the groups was non-significant (*p* > 0.05).

**Table 1:** Mean (SD) surface roughness in Sa values before and after scaling with PS or conventional scaler tips

Surface roughness	Mean (SD) µm		Mean difference (SD)	*p* value	*p* value
	Before	After			
PS (n = 10)	9.8 (4.7)	6.7 (3.3)	3.1 (2.5)	0.005*	0.167
C (n = 10)	10.0 (3.2)	5.3 (3.5)	4.7 (3.3)	0.005*	

Intragroup comparison was analysed using Wilcoxon signed-rank test. Intergroup comparison was analysed with independent t test

*C* conventional tip, *PS* Perio Slim tip, *SD* standard deviation

*Statistically significant difference (*p* < 0.05)

#### Tooth substance loss

**Fig. 7:**
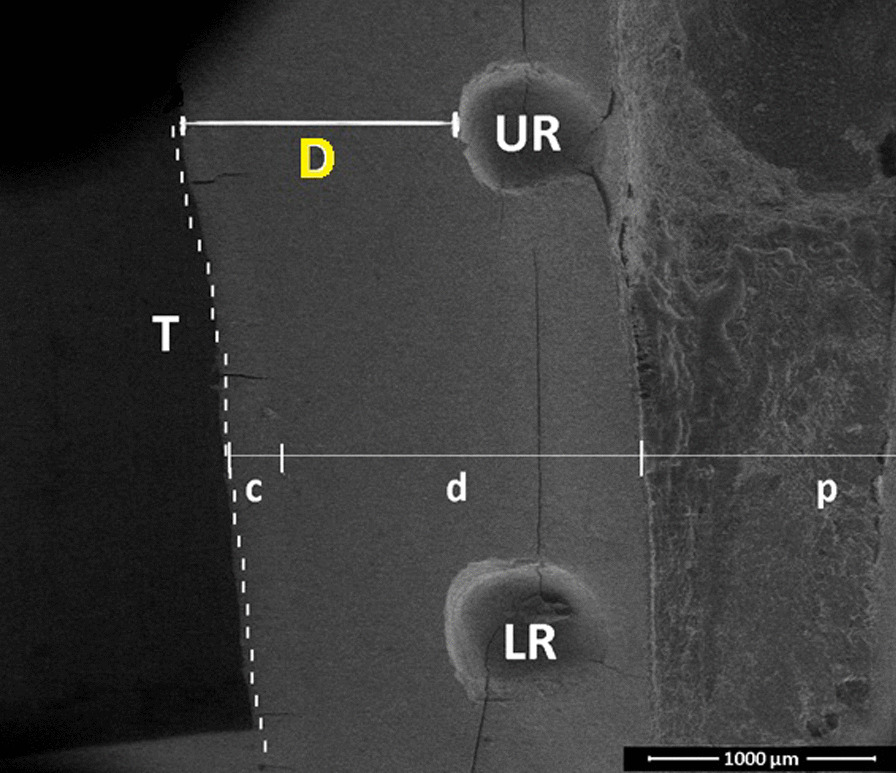
Scanning electron micrograph showing a cross-section of the tooth before scaling at × 50 magnification. Two reference points shown: upper reference point (UR) and lower reference point (LR). Tooth thickness (D) is measured from the outer tooth surface (T) (dotted lines) to the reference points, UR and LR. c is cementum, d is dentine and p is pulp

The distance (D) between the outer tooth surface (T) and upper reference point (UR) or lower reference point (LR) is considered the ‘tooth thickness’. Tooth thickness before scaling is referred to as ‘thickness before,’ and tooth thickness after scaling is referred to as ‘thickness after’. Figure 7 shows representative scanning electron micrograph of cross-section of a tooth.

Mean (SD) of tooth thickness (µm) before and after scaling at upper and lower reference points are shown in Tables 2 and 3. Findings on tooth thickness was categorised into < 1000 µm and ≥ 1000 µm. The categorisation was performed due to the differences in the microhardness between tooth layers i.e. cementum, outer dentine, middle dentine, inner dentine [24] and its possible influence on the degree of tooth substance loss. In this study, there was no identification of tooth layers therefore, the categorisation seems crucial. Middle dentine is the hardest layer compared to cementum, inner and outer dentine layers [24]. In cases where all the layers are intact (more than 1000 µm), it is anticipated that there should be more tooth substance loss since the outer layers (cementum and outer dentine) are soft. In contrast, if the thickness is less than 1000 µm, it is anticipated that there will be more tooth substance loss following scaling since the middle dentine is the hardest among all.

**Table 2:** Tooth substance loss (µm) at upper and lower reference points before and after scaling with PS or conventional scaler tips in teeth with initial thickness of < 1000 µm

Reference point	Scaler tip	Mean µm (SD)		Mean difference (SD)	*p* value	*p* value
		Before	After			
UR	PS (*n* = 9)	790.9 (130)	778.7 (130)	12.2 (8)	0.002*	0.038*
	C (*n* = 8)	745.0 (197)	724.8 (212)	20.2 (25)	0.058	
LR	PS (*n* = 9)	811.9 (179)	796.0 (178)	16.0 (13)	0.007*	0.0375*
	C (*n* = 8)	802.4 (269)	780.5 (280)	21.9 (16)	0.007*	

Intragroup comparison was analysed with paired *t* test. Intergroup comparison for UR and LR was analysed with independent sample *t* test. UR for upper reference point, and LR for lower reference point

*C* conventional, *PS* Perio Slim

*Statistically significant different (*p* < 0.05)

**Table 3:** Tooth substance loss at upper and lower reference point before and after scaling using PS or conventional scaler tips among teeth with initial thickness ≥ 1000 µm

Reference point	Scaler tip	Mean µm (SD)		Mean difference	*p* value	*p* value
		Before	After			
UR	PS (n = 14)	1280.1 (190)	1265.8 (189)	14.3 (10)	0.0001^*^	0.058
	C (n = 15)	1267.9 (121)	1223.7 (113)	44.2 (51)	0.005^*^	
LR	PS (n = 14)	1131.0 (25)	1113.0 (254)	17.3 (11)	0.001^*^	0.16
	C (n = 15)	1218.0 (200)	1177.0 (191)	41.3 (49)	0.006^*^	

Intragroup comparison analysed with paired t test. Intragroup comparison for LR (PS) analysed with Wilcoxon signed-rank test. Intergroup comparison for UR and LR was analysed with Mann–Whitney test

*UR* upper reference point, *LR *lower reference point, *C *conventional, *PS* Perio Slim

^*^Statistically significant difference (*p* < 0.05).

### Initial tooth thickness < 1000 µm

*At the upper reference point*, the mean thickness before scaling was 790.9 (130) µm and 745.0 (± 197) µm in the PS and conventional group, respectively (Table 2). After scaling, thickness decreased to 778.7 (± 130) µm and 724.8 (± 212) µm in the PS and conventional groups, respectively. The mean difference of tooth thickness following scaling was significantly low in the PS group (*p* < 0.05).

*At the lower reference point*, the mean thickness before scaling was 811.9 (± 179) µm and 802.4 (± 269) µm in the PS and conventional group, respectively. After scaling, thickness decreased to796.0 (± 178) and 780.5 (± 280) µm in the PS and conventional groups, respectively. The mean difference in tooth thickness after scaling was significantly lower in the PS group than that in the conventional group when the initial thickness was < 1000 µm (*p* < 0.05).

### Initial thickness of ≥ 1000 µm

*At the upper reference point*, the mean thickness before scaling was 1280.1 (± 190) and 1267.9 (± 121) µm in the PS and conventional groups, respectively (Table 3). After scaling, the mean thickness decreased to 1265.8 (± 189) and 1223.7 (± 113) µm in the PS and conventional groups, respectively. After scaling, the mean difference between PS and conventional groups was not significant (*p* > 0.05).

*At the lower reference point*, the mean thickness before scaling was 1131.0 (± 25) and 1218.0 (± 200) in the PS and conventional groups, respectively. After scaling, the thickness decreased to 1113.0 (± 254) and 1177.0 (± 191) µm in the PS and conventional groups, respectively. The mean difference in tooth thickness after scaling between the PS and conventional groups was not significant (*p* > 0.05).

#### Clinical study

Table 4 summarises the socio-demographic characteristics of the participants in group A (n = 15) and group B (n = 15). The number of males was slightly higher than that of females in both groups, but the difference was not significant. The majority of the participants belonged to Malay ethnicity. For both groups, the majority of the participants (80%) were in the 20–30 year age range. Almost all participants (93%-100%) had at least tertiary education. There was no statistically significant difference between group A and group B with regards to gender, ethnicity, age, and level of education (*p* > 0.05).

**Table 4:** Socio-demography characteristics of participants in group A and group B

Characteristics	Group A (*n* = 15) *n* (%)	Group B (*n* = 15) *n* (%)	*p* value
*Gender*			
Male	8 (53)	9 (60)	0.71
Female	7 (47)	6 (40)	
*Ethnicity*			
Malay	13 (87)	14 (93)	0.50
Others	2 (13)	1 (7)	
*Age*			
20–30	12 (80)	12 (80)	0.72
31–40	3 (20)	3 (20)	
*Level of education*			
Primary	0 (0)	0 (0)	0.32
Secondary	1 (7)	0 (0)	
Tertiary	14 (93)	15 (100)	

Group A: Perio Slim PS scaler tip at Q1 followed by Conventional scaler tip at Q2. Group B: Conventional scaler tip at Q1 followed by Perio Slim PS scaler tip at Q2. Intergroup comparison was analysed using Mann–Whitney test

The baseline periodontal parameters are summarised in Table 5. The mean PPD was 2.76 ± 0.18 mm and 2.77 ± 0.23 mm for PS and conventional groups, respectively. No statistically significant difference between the PS and conventional groups with regards to PPD, CAL, GBI and VPI (*p* > 0.05).

**Table 5:** Baseline periodontal parameters comparison based on the type of scaler tip used; Perio Slim (PS) or Conventional

Clinical parameters	Mean (SD)			
	PPD (mm)	CAL (mm)	GBI (%)	VPI (%)
PS (*n* = 30)	2.76 (0.18)	2.96 (0.22)	0.57 (0.17)	0.47 (0.13)
C (*n* = 30)	2.77 (0.23)	2.99 (0.23)	0.56 (0.18)	0.50 (0.24)
*p* value	0.60	0.68	0.67	0.79

Intergroup comparison was analysed using paired sample *t* test

*C* conventional, *PPD* mean probing pocket depth, *CAL* mean clinical attachment level, *GBI* mean gingival bleeding index, *VPI* mean visible plaque index

**Fig. 8:**
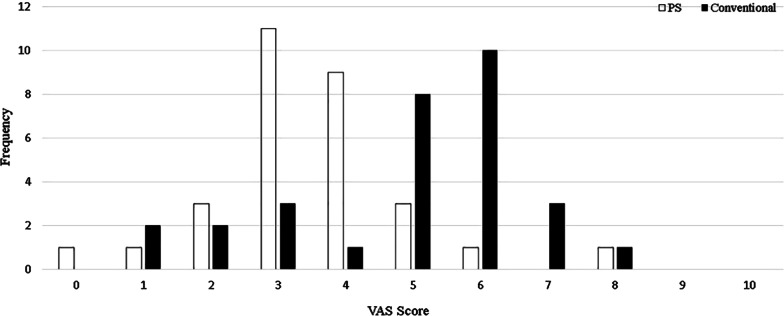
Frequency distribution of VAS scores for Perio Slim (PS) and conventional scaler tips

Figure 8 shows the frequency distribution of VAS scores for PS and conventional scaler tips. The most frequent score for PS group was 3, but for conventional group was 6.

Table 6 summarises the mean pain scores for PS and conventional groups. Mean pain scores were 3.5 (± 1.5) and 4.9 (± 1.8) for PS and conventional groups, respectively. The median pain scores were 3 for the PS group and 5 for the conventional group. Pain score was significantly higher in the conventional group (*p* < 0.05).

**Table 6:** Mean (standard deviation) and median (IQR) pain scores comparison between Perio Slim (PS) and conventional scaler tips

Scaler tip	VAS score		
	Mean (SD)	Median (IQR)	*p* value
PS	3.5 (1.5)	3 (1)	0.003*
Conventional	4.9 (1.8)	5 (2)	

Intergroup comparison was analysed using Wilcoxon signed-rank test

*Statistically significant difference (*p* < 0.05)

## Discussion

Over the last few years, new scaler tip designs have been introduced for painless and less aggressive scaling. Given that periodontal treatment success is largely dependent on continuous periodontal visits, repeated instrumentation may expose tooth structures to risks of tooth substance loss and tooth surface roughness. Moreover, patients undergoing life-long supportive periodontal therapy may have been affected in view of their compliance if treatment was perceived as painful.

To the best of our knowledge, no previous study compared tooth surface roughness produced by piezoelectric ultrasonic scalers using either slim (Perio Slim) or conventional scaler tips. An earlier study investigated surface roughness outcomes on restorative materials; the conventional scaler tip caused rougher surfaces than the slim scaler tip (21). In the current study, the effect of scaler tip design on tooth surface roughness was not significantly different possibly because of the manufacturer’s setting that were meant to be gentle or non-aggressive to tooth substance. Regardless of design, our study used new scaler tips because worn scaler tips increase tooth surface roughness [22, 23]. For all samples, similar new scaler tip was used. According to the manufacturer, the scaler tip will only be defined as worn after 100 cycles of use. Thus, a non-aggressive standardised scaling protocol and brand-new scaler tips accounted for the non-significant difference in tooth surface roughness between the two scaler tip designs.

The study also reported that when the initial tooth thickness was < 1000 µm, tooth substance loss was lower when scaling was carried out with a PS scaler tip than when a conventional scaler tip was used. A thinner tooth structure may comprise less calcified dentine, which constitutes the hard part of the subgingival portion of a tooth structure. The middle layer of root dentine was harder than the cementum and other layers of root dentine [24]. In cases where all the layers are intact (> 1000 um), it is anticipated that there should be more tooth substance loss since the outer layers (cementum and outer dentine) are soft. In contrast, if the thickness is < 1000 um, it is anticipated that there will be more tooth substance loss following scaling since the middle dentine is the hardest among all.

The limitation of the study includes lack of information on the history of the extracted teeth whether the teeth were exposed to repeated scaling which causes root cementum loss. Besides, identification of cementum and dentine layers was not performed due to source and time constraint. In a clinical setting, the layers of root substance cannot be determined. Given that periodontal patients are exposed to risks of tooth substance loss after repeated instrumentation, using a slim scaler tip design is an advantage.

Slimmer scaler tip design was subjected to the dampening effect that produces flattened oscillation pattern by which displacement amplitude is reduced [4]. The flattened oscillation pattern reduces impact on tooth surface and can be the reason that the reduced tooth substance loss from PS scaler tips.

The reduced tooth substance loss after the use of PS scaler tip compared with conventional scaler tip was not due to the differences in scaling procedure. Standardised scaling procedure was practised in this study under a medium power setting, light force, parallel technique and standardised duration of 2 min. Increased tooth substance loss is associated with increasing tip angulation, power setting and force [4, 10, 25].

The findings of this study were in agreement with Jepsen et al. [13], where slim scaler tips caused less tooth substance loss compared to wider scaler tips. Kawashima et al. [9] reported higher Roughness Loss of Tooth Substance Index [26] score with the use of hand curette compared to Vector scalers [4]. Vector produced a linear oscillating pattern which is similar to the PS scaler tip oscillation pattern [10].

In the current study, the operator was not blinded to the scaler tip design. Thus, risk of bias in terms of pressure exerted during scaling. Flemmig et al. [25] reported that there was no significant difference in the amount of root damage following scaling with lateral pressure of 0.5 N compared to 1.0 N, when parallel technique was used. Parallel technique is defined as zero-degree angulation between scaler tip and the tooth surface.

The findings from the clinical part of the present study demonstrated that PS scaler tip design caused less pain during scaling than a conventional scaler tip. These findings are in agreement with a previous study by Braun et al. [17]. Braun et al. [17] compared the subjective pain intensity during ultrasonic scaling (Sirosonic L, Sirona, Germany) between conventional scaler tip (Instrument No. 3, Sirona, Germany) and slim-line style (Perio Pro Line Instrument SI-11, Sirona, Germany) scaler tip through an intermodal intensity technique. Pain sensation was less when using slim compared to conventional scaler tip, with a median pain score of 1.4 U and 7.8 U for slim-line and conventional scaler tips, respectively [17].

Pain during scaling could be due to the oscillation pattern produced by the vibration of the scaler tip. Less pain following scaling using PS scaler tip could be attributed to the flattened oscillation pattern [20]. Research on the relationship between displacement amplitude and pain level could further clarify the causes of pain during scaling procedure.

In order to reduce the differences in pain threshold between participants, a split-mouth study design was used in this study and it was commonly reported in other studies [17, 27, 28]. The limitation of this study design is that there is a possibility that the patient may still remember the pain experienced from the first scaling. Subsequently, this could influence the second pain score because of the desensitisation caused by repeated exposure [29]. However, a wash-out period of one-hour before the second procedure could reduce the desensitisation effect.

The study indicates that for periodontal patients that have had repeated scaling and left with minimal and/or delicate tooth structure, the use of slim design ultrasonic scaler tips is patient-friendly. This will be in line with the overall aim to deliver dental care with a minimum patient discomfort. Thus, increasing a patient’s compliance during dental treatment may be possible.

Other limitation in the in vitro study includes small sample size. On the other hand, for the clinical study, other limitations includes clinician was not blinded and no correlation between pain and treatment time was carried out.

## Conclusion

Within the limitation of the study, it was demonstrated that in the in vitro study, using a slim scaler tip design causes less tooth substance loss compared to a wide (conventional) scaler tip design. In the clinical study, less pain was observed compared than a wide (conventional) scaler tip design.

## Data Availability

All data generated or analysed during this study are included in this published article.

## Abbreviations

PS: Perio Slim
VAS: Visual analogue scale
PPD: Probing pocket depth
CAL: Clinical attachment level
GBI: Gingival bleeding index
VPI: Visible plaque index
IQR: Interquartile range
SD: Standard deviation
SEM: Scanning electron microscope
